# A Space-Time Network-Based Modeling Framework for Dynamic Unmanned Aerial Vehicle Routing in Traffic Incident Monitoring Applications

**DOI:** 10.3390/s150613874

**Published:** 2015-06-12

**Authors:** Jisheng Zhang, Limin Jia, Shuyun Niu, Fan Zhang, Lu Tong, Xuesong Zhou

**Affiliations:** 1 School of Traffic and Transportation, Beijing Jiaotong University, Beijing 100044, China; E-Mail: zjs2107@163.com; 2 Research Institute of Highway Ministry of Transport, No. 8 Xitucheng Rd., Haidian District, Beijing 100088, China; E-Mails: nsy@itsc.cn (S.N.); zhangfan@itsc.cn (F.Z.); 3 State Key Laboratory of Rail Traffic Control and Safety, Beijing Jiaotong University, No. 3 Shangyuancun, Haidian District, Beijing 100044, China; E-Mail: lmjia@vip.sina.com; 4 School of Traffic and Transportation, Beijing Jiaotong University, No. 3 Shangyuancun, Haidian District, Beijing 100044, China; E-Mail: ltong@bjtu.edu.cn; 5 School of Sustainable Engineering and the Built Environment, Arizona State University, Tempe, AZ 85287, USA

**Keywords:** unmanned aerial vehicle, traffic sensor network, space-time network, lagrangian relaxation, route planning

## Abstract

It is essential for transportation management centers to equip and manage a network of fixed and mobile sensors in order to quickly detect traffic incidents and further monitor the related impact areas, especially for high-impact accidents with dramatic traffic congestion propagation. As emerging small Unmanned Aerial Vehicles (UAVs) start to have a more flexible regulation environment, it is critically important to fully explore the potential for of using UAVs for monitoring recurring and non-recurring traffic conditions and special events on transportation networks. This paper presents a space-time network- based modeling framework for integrated fixed and mobile sensor networks, in order to provide a rapid and systematic road traffic monitoring mechanism. By constructing a discretized space-time network to characterize not only the speed for UAVs but also the time-sensitive impact areas of traffic congestion, we formulate the problem as a linear integer programming model to minimize the detection delay cost and operational cost, subject to feasible flying route constraints. A Lagrangian relaxation solution framework is developed to decompose the original complex problem into a series of computationally efficient time-dependent and least cost path finding sub-problems. Several examples are used to demonstrate the results of proposed models in UAVs’ route planning for small and medium-scale networks.

## 1. Introduction

Reliable and timely traffic information is the foundation of network-wide traffic management and control systems. An advanced traffic sensor network needs to rapidly detect non-recurring traffic events and reliably estimate recurring traffic congestion along key freeway and arterial corridors. Most of commonly used traffic sensors are equipped at fixed locations, such as loop detectors, microwave detectors, video cameras, Automatic Vehicle Identification (AVI) readers, *etc*. Fixed traffic sensors can constantly monitor traffic dynamic characteristics of the specific location for a long time horizon, but they cannot provide a full spatial and temporal coverage in a network due to construction and maintenance budget constraints. With the use of movable or mobile traffic sensors, the next-generation transportation sensor network is expected to offer a more reliable and less costly approach to rapidly detect complex and dynamic state evolution in a transportation system.

Our research will focus on how to integrate existing fixed and emerging mobile sensors into a dynamic traffic monitoring system that can significantly improve spatial coverage responsiveness to important events. Specifically, the new generation of small Unmanned Aerial Vehicles (UAVs) now offers outstanding flexibility as low-cost mobile sensors. UAVs can be launched quickly and exchange data with the control center in real time by using wireless transmission systems. While in the last decade UAVs have been widely used in the military field, UAVs are still facing some technical and institutional barriers in civilian applications, for instance, strict airspace and complicated route restrictions. Recently, many countries, such as the United States and China, have begun considering and evaluating flexible air traffic control rules that allow the low attitude space (lower than 1000 m) management for UAV-based civil engineering applications, such as, traffic detection, weather monitoring, disaster response and geological survey. This emerging trend presents a great opportunity for the transportation agencies and operators to explore the full potential of UAVs in road traffic network surveillance and traffic incident monitoring. The common equipped sensors on the UAVs can produce entire images of an investigation area or a special location, which can be further post-processed to monitor semi-continuous traffic state evolution. In this research, we are interested in developing computationally efficient optimization models for using UAVs to improve the effectiveness of traffic surveillance in conjunction with traditional fixed traffic sensors.

To rapidly and reliably capture traffic formation and congestion on the traffic network, a dynamically configured sensor network should be able to recognize time-varying traffic flow propagation that expands to both space and time dimensions. In this research, we adopt a modeling approach from the time-geography field [1,2], in order to systematically take into account both geometry and topology of the road network and time attributes of each event along UAV cruising routes. The particular area of interest for this UAV dynamic route planning application is how to rapidly enable road traffic and incident monitoring. Based on a linear programming model and a space-time network characterization, we develop an UAV routing/scheduling model which is integrated with existing fixed traffic monitoring sites for road segments with various frequencies of incidents. The goal of our model is to minimize the total cost in terms of the detection delay of spatially and temporally distributed incidents by both fixed sensors and UAVs. The total budget and UAVs’ feasible routing routes are also considered as practical constraints in our model. To address the computational efficiency issue for real-world large scale networks, a Lagrangian relaxation method is introduced for effective problem decomposition.

The remainder of this paper is organized as follows: a literature review and problem statements are presented first in the next section. In Section 3, a space-time network-based UAV routing planning model is developed to integrate with existing fixed traffic detectors to maximize spatial and temporal coverage. Section 4 further presents the Lagrangian relaxation solution algorithmic framework, followed by several illustrative examples and numerical experiment results to demonstrate the effectiveness of the proposed models in Section 5.

## 2. Literature Review

### 2.1. Related Studies

On-line applications of intelligent traffic network management call for the reliable detection, estimation and forecasting of dynamic flow states so that proactive, coordinated traffic information and route guidance instructions can be generated to network travelers for their pre-trip planning and en-route diversion. The problem of how to optimize traffic sensor locations to maximize the spatial coverage and information obtainable has been extensively studied by many researchers. Gentili and Mirchandani [3] offered a comprehensive review for three different sensor location optimization models (sensor type, available a-priori information and flows of interest), and classified them into two main problems: the observability problem and the flow-estimation problem. A partial observability problem is also studied by Viti *et al*. [4]. For origin-destination demand and estimation applications, many sensor location methods are based on the study by Yang and Zhou [5] which focuses on how to maximize the coverage measure in terms of geographical connectivity and OD flow demand volume. For travel time estimation, Sherali *et al*. [6] proposed a quadratic binary optimization model for locating AVI readers to capture travel time variability along specified trips. A dynamic programming formulation was developed by Ban *et al*. [7] to minimize link travel time estimation errors through locating point sensors along a corridor. In the reliable sensor location problem studied by Li and Ouyang [8], an integer programming model is developed to consider random sensor failure events. Based on a Kalman filtering framework, Xing *et al*. [9] extended an information-theoretic modeling approach from Zhou and List [10] for designing heterogeneous sensor networks in travel time estimation and prediction applications. It should be noted that, the water network sensor placement problem is also closely related to the problem of traffic sensor network design, as many studies such as that of Berry *et al*. [11] focus on how to improve spatial coverage and event detectability for water pollution sources, by adapting p-median location models.

UAV systems have been used as emerging mobile monitoring tools to conduct different tasks by loading various sensors, such as high-resolution camera, radar, and infrared camera. There are a wide range of UAVs studies for different transportation domain applications. For instance, by using high-resolution images from UAVs, the Utah Department of Transportation examined how to improve their highway construction GIS databases [12]. The Florida Department of Transportation studied the feasibility of using surveillance video from UAVs for traffic control and incident management [13]. Recently, Hart and Gharaibeh [14] examined the use of UAVs for roadside condition surveys.

It is also widely recognized that, there are still a number of limitations for using UAVs in civilian transportation applications. First, the accuracy of traffic information collection depends on weather conditions and specific types of sensors carried by UAV. The maximum flight distance or flight time of UAV is constrained by the fuel weight and number of battery units. If the number of available UAVs is given, it is important to optimize the cruise route plan of UAVs in order to cover more roads of interest under the UAV capacity constraints. Ryan *et al*. [15] considered this UAV routing problem as a multiple Travel Salesman Problem (TSP) with the objective of maximizing expected target coverage and solved it by applying a Reactive Tabu Search. In the study by Hutchison [16], the monitored roads are divided into several sub-areas first, and then all the selected roads in each sub-area is covered by one UAV, equipped with a simulated annealing algorithm. Yan *et al*. [17] also considered the UAVs routing problem as a multi-vehicle TSP and introduced a generic algorithm to design close-to-optimal routes to consider different flight paths between two target roads.

In the area of collaborative UAV route planning, the method proposed by Ousingsawat and Campbell [18] first finds the shortest path between two points, and then solves the corresponding task assignment problem. Tian *et al*. [19] introduced the time window requirement of reconnaissance mission for each target road, and considered the constraints of maximum travel time of each UAV through a Genetic Algorithm. In the study by Wang *et al*. [20], a multi-objective ant colony system algorithm is used for UAV route planning in military application with both route length and danger exposure being minimized in the cost functions. The multi-objective optimization model by Liu *et al*. [21] aims to minimize the total distance and maximize the total number of monitored points subject to the available number of UAVs and maximum cruise distance constraints. The model is solved by the genetic algorithm to search for satisfactory UAV routes. The studies by Liu *et al*. [22] and Liu *et al*. [23] introduce a time window constraint and examine UAV route planning methods without and with flight distance constraints. They used a K-means clustering algorithm to decompose the UAV cursing area into a number of sub-areas, and further applied a simulated annealing-based solution algorithm. The multi-objective optimization model proposed by Liu *et al*. [24] aims to minimize UAV cruise distance and minimize the number of UAVs being used.

Mersheeva and Friedrich [25] adopted a metaheuristic variable neighborhood search algorithm, and Sundar and Rathinam [26] presented a mixed integer programming model for UAV route planning with refueling depot constraints. A recent study by Ning *et al*. [27] specifically considers the mobility constraints of traffic sensors, and a measure of traffic information acquisition benefits was used to evaluate the surveillance performance. Their proposed hybrid two-stage heuristic algorithms include both particle swarm optimization and ant colony optimization components. Table 1 offers a systematic comparison for the literature reviewed in Section 2.1.

**Table 1:** Summary of existing UAV route planning studies.

Paper	Model and Formulation	Solution Algorithm	Factors under Consideration
Ryan *et al*. (1998) [15]	Multi-vehicle Traveling Salesman Problem	Reactive tabu search heuristic within a discrete event simulation	Target coverage, time window
Hutchison (2002) [16]	Two-stage model for problem decomposition and single-vehicle TSP problem	Simulated annealing method	Target coverage, UAV flight distance
Yan *et al*. (2010) [17]	Multi-vehicle TSP problems	Genetic algorithm	Flying direction on each link
Ousingsawat and Campbell (2004) [18]	Cooperative reconnaissance problem	A* search and binary decision	Maximum time duration, target coverage, UAV conflicts
Tian *et al*. (2006) [19]	Cooperative reconnaissance mission planning problem	Genetic algorithm	Maximum time duration , UAV conflict, time window
Wang *et al*. (2008) [20]	Multi-objective optimization model	Ant colony system algorithm	Minimum length and threat intensity of the path
Liu, Peng, Zhang (2012) [21]	Multi-objective optimization model	Non-dominated sorting genetic algorithm	Number of UVAs
Liu, Peng, Chang, and Zhang, (2012) [22]	Multi-objective optimization model	Multi-objective evolutionary algorithm	Time window
Liu, Chang, and Wang (2012) [23]	Traveling Salesman Problem	Simulated annealing method	Target coverage, number of UAVs
Liu, Guan, Song, Chen, (2014) [24]	Multi-objective optimization model	Evolutionary algorithm based on Pareto optimality technique	UAV flight distance, number of UAVs
Mersheeva and Friedrich (2012) [25]	Mixed-integer programming model	Variable neighborhood Search	Target coverage, UAV flying time
Sundar and Rathinam (2012) [26]	Single-vehicle UAV routing problem	mixed integer, linear programming	Target coverage, refuel depot
This paper by Zhang *et al*.	Linear integer programming model within space-time network	Problem decomposition through Lagrangian relaxation and least cost shortest path algorithm for subproblems	Detecting recurring, non-recurring traffic conditions and special events

While a large number of studies have been devoted to the UAV route planning problem with general spatial coverage measures, the potential benefits of utilizing UAVs to capture traffic propagation in both space and time dimensions have not been adequately exploited, especially for cases with stochastic non-recurring traffic incidents with large impacts on traveler delay and route choice. Most of the existing research only focuses on the static monitoring coverage measure, while it is critically needed to adopt a systematic space-time coverage measure for the integrated sensor network design problem with both fixed and mobile sensors. For the VRP problem focusing on UAV routing in a traffic network, the corresponding theoretical and algorithmic aspects along this line are still relatively undeveloped, and these challenging questions calls for flexible and systematic modeling methodologies and efficient solution algorithms for large-scale network applications.

### 2.2. Illustrations of Conceptual Framework for Space-Time Networks

**Figure 1:**
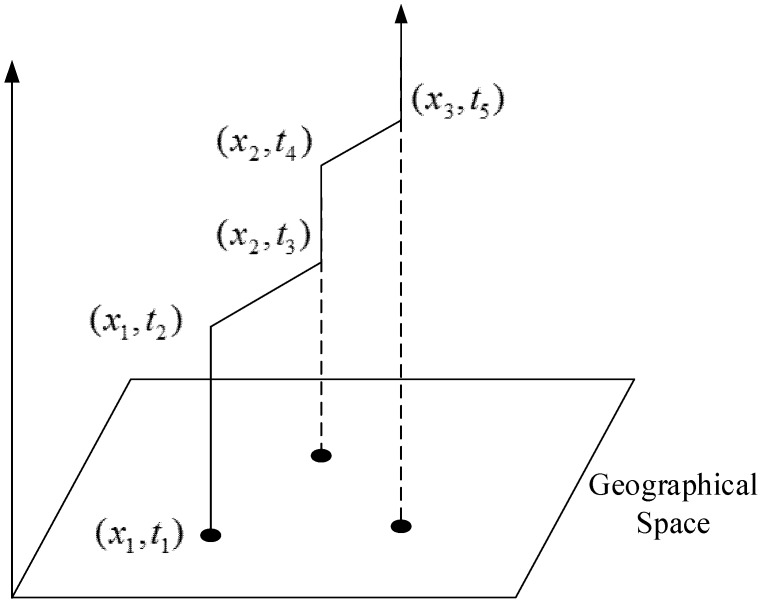
Illustration of a space-time path.

In this paper, a time geography based modeling approach is adopted for the traffic sensor network design problem. This theory is introduced by Hagerstrand [1] to specifically use space-time paths and space-time prisms in accessibility assessment. A space-time path represents the path taken by an individual agent in a continuous space, with a travel time budget constraint. A space-time prism is the set of all points that can be reached by an individual, given a maximum possible speed from a starting point and an ending point in space-time [2,28]. A simple space-time path example is illustrated in Figure 1.

The agent (*i.e.*, UAV in our research) starts from location *x*_1_ (as an UAV depot) at time *t*_1_ and departs from *x*_1_ to *x*_2_ (e.g., incident site) at *t*_2_; at time *t*_3_, the agent arrives at *x*_2_ and stays at *x*_2_ until *t*_4_ (to monitor the traffic delay), then the agent moves towards location *x*_3_ and arrives there at time *t*_5_. The different slopes represent different flying speeds in our example.

**Figure 2:**
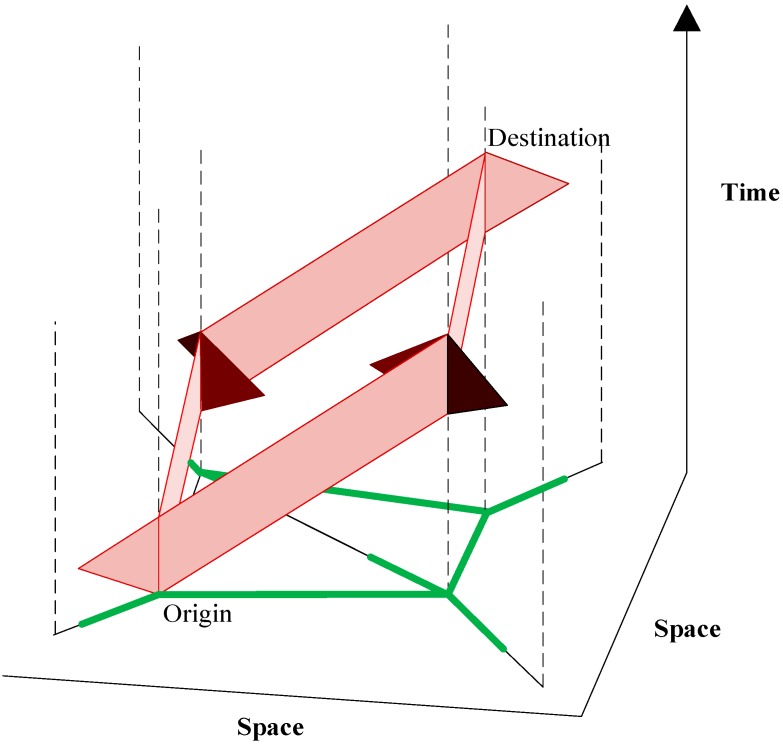
Constrained space–time prism (red) on road networks (green and black) and its spatial projection (green). Adapted from [28].

A space-time prism is an important concept for analyzing travelers’ accessibility on the transportation network and it is used in many transportation planning studies. Utilizing this space-time prism concept in the UAV routing planning application, we can also clearly examine the relationship between geographic space and time horizon. The accessibility or reachability by an UAV sensor can be represented by the prism volumes. In order to consider more spatial constraints within a real-world road network, such as connectivity, speed range, and geometry, a network-space prism concept can be used. Figure 2 from Kuijpers and Othman [28] shows a network time prism in network-time space (shown in red regions) and its potential paths’ projection to road networks (shown in green). The green projection on the physical network represents individual’s potential traveling routes from the origin to the destination.

**Figure 3:**
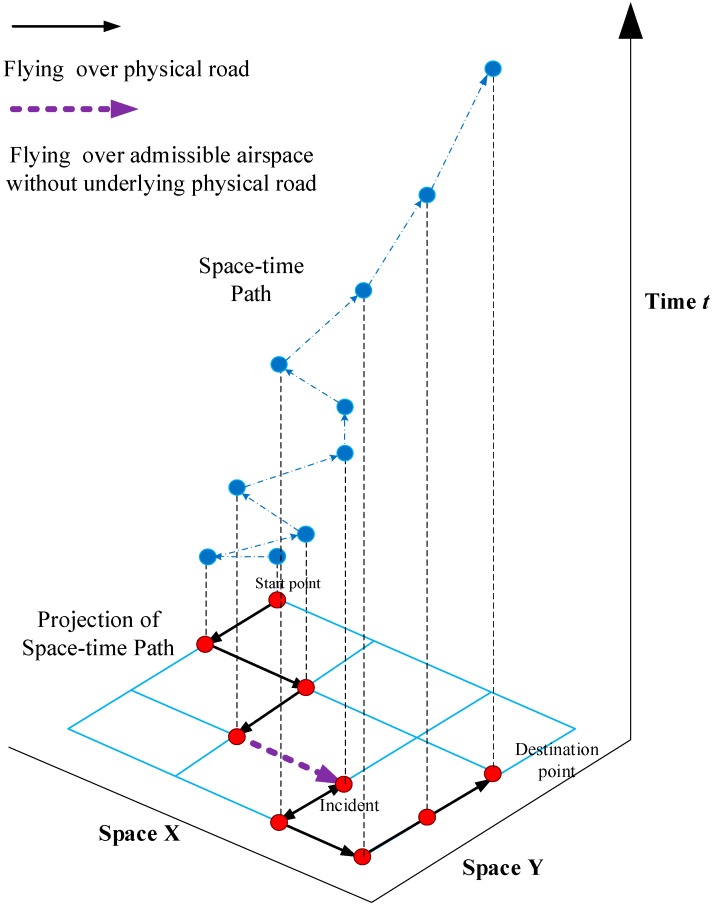
UAV routing path in a discretized space-time network.

Figure 3 shows an UAV trajectory in the context of network-time paths. With regards to the admissible air space and flying speed considerations, the feasible UAV’s routes could be predefined along the physical road network and possible air space discretized in both space and time. In Figure 3, the blue lines are the physical network links; black lines are the UAV route on physical links; purple dash line is the UAV route on admissible air space.

**Figure 4:**
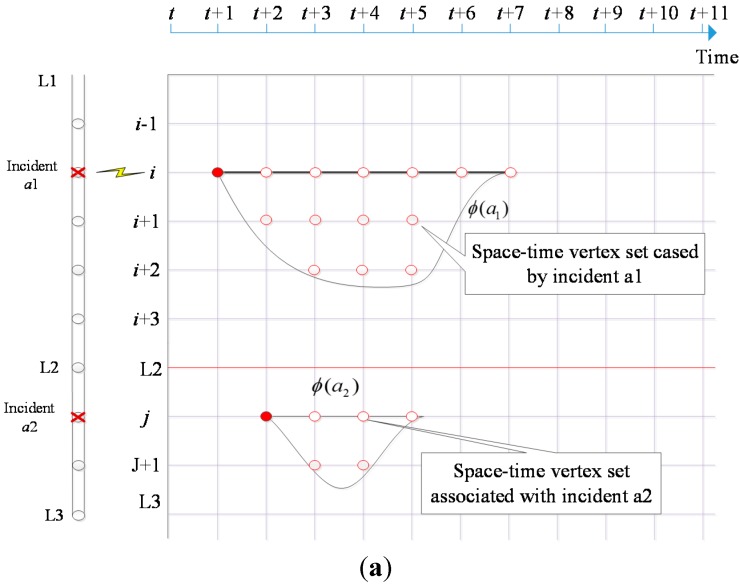

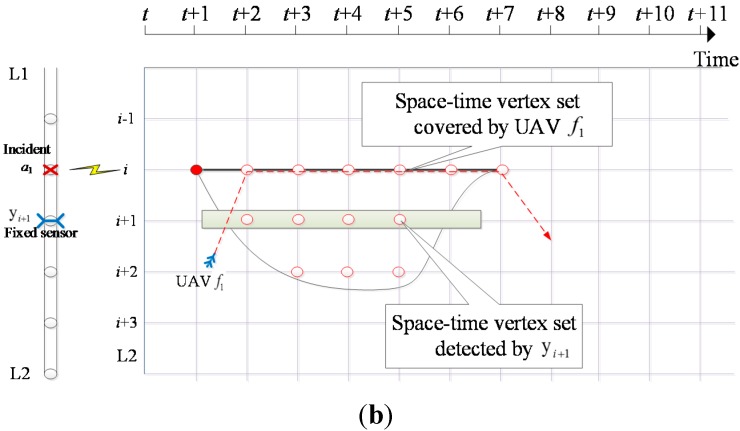
(**a**) Sets of space-time vertexes affected by different events *a*1, and *a*2; (**b**) Set of space-time vertexes detected by fixed sensor and UAV.

With a discretized space-time network construct, one of the remaining challenging issues is how to maximize the traffic information obtainable at strategically critical locations and time-sensitive durations within the UAV traveling budget constraint in conjunction with the existing fixed sensor detection infrastructure. We assume that there are probabilistic traffic incident event data, obtainable from historical data or observed directly from a real time environment. With the time as the horizontal axis, Figure 4a shows the space-time feature of traffic incident, where the congestion due to the traffic incident is propagated and dissipated along the corridor as the time advances. Accordingly, one can define a space-time vertex set denoted as *ϕ*(*a*), for each incident event *a* at a location. The road segments with very low incident rates typically need to be patrolled once or twice a day in order to find soft time windows with low priority for the UAVs. To better consider the UAV speed and altitude restrictions in further research, one can create a multi-dimensional model, where each vertex is characterized by the longitude, latitude and altitude at different time stamps, and accordingly limit the feasible route search space by considering the vertexes satisfying altitude restrictions and the arcs satisfying speed requirements.

Ideally the entire space-time vertex in a set should be fully observed by either fixed traffic sensors or UAVs at any given time, which means that the incident and its impact area are fully detected. Without loss of generality, this paper assumes that, if a fixed traffic sensor is located on a site inside the vertex set of *ϕ*(*a*), then this sensor can cover all the space-time vertex on this location at all time. If an UAV flies to site *i* at time *t* + 1, as shown in Figure 4b then the space-time vertex (*i*, *t* + 1) is marked as covered, if it stays at this site for five time periods, then all six out of the seven total space-time vertexes on site *i* are observed with 1 time unit of detection delay.

## 3. Model Description

### 3.1. Notations

We first introduce some key notations used in the UAV routing planning problem.

Parameters are shown in Table 2:

**Table 2:** Subscripts and parameters used in mathematical formulations.

Symbol	Definition
A	set of traffic accidents/events
N	set of nodes in transportation network
V	set of space-time vertexes
E	set of space-time traveling arcs that UAV can select
F	set of UAVs
a,a′	indices of traffic accidents/events, a,a′∈A
i,j	indices of candidate sensor locations or possible UAV locations, i,j∈N
t, s	indices of time stamps
(i,t),(j,s)	indices of space-time vertexes
(i,j,t,s)	index of space time traveling arc, (i,j,t,s)∈E
f	index of unmanned plane, f∈F
$o^{f},\;d^{f}$	indices of origin and destination locations of UAV f
$EDT^{f},\;LAT^{f}$	earliest departure time and latest arriving time of UAV f
T(a)	start time of event a
ϕ(a)	set of space-time vertexes which can represent the space-time impact area of event a
ϕ′(a)	subset of time-space vertex set ϕ(a) which excludes the space time vertexes covered by the fixed detectors. ϕ′(a)⊆ϕ(a)
$d_{i,j,t,s}$	cost for UAVs to travel between location *i* and location *j*, on time period between time *t* and time *s*
*d_i_*	cost for constructing a fixed sensor at location *i*
$c_{i,t}^{F}(a)$	fail-to-detect cost of event *a* at location *i* and time t
*B*	total budget for constructing fixed sensors
*K*(*f*)	total distance budget for operating UAV *f*

Variables are shown in Table 3:

**Table 3:** Decision variables used in mathematical formulations.

Symbol	Definition
$x_{i,t}(a)$	event detected variable (= 1, if event *a* is detected at location *i*, time *t*; otherwise = 0)
${x^{F}}_{i,t}(a)$	event virtually detected variable (= 1, if event *a* is virtually detected at location *i*, time *t*; otherwise = 0)
$y_{i}$	fixed sensor construction variable (= 1, if fixed sensor is allocated at location *i*; otherwise = 0)
$w_{i,j,t,s}(f)$	UAV routing variable (= 1, if space-time arc (*i*,*j*,*t*,*s*) is selected by UAV *f*; otherwise = 0)

In this paper, we do not assume a constant cruising speed of Ubetween a node pair (*i*, *j*). Instead, we allow different travel times (denoted as time *t* to time *s*) between a link (*i*, *j*) to reflect different flying speed, corresponding to various degrees of fuel consumptions.AV can also stay at the node for an extended time period within the regulation constraints, denoted as a staying arc (*i*, *i*, *t*, *t* + 1) at the same node *i*. In addition, the virtual sensors are introduced in our model to capture the cost loss of non-coverage. Thus, we assume that every point at a vertex set within the incident impact area could be monitored by either fixed sensors, UAVs or virtual sensors. Accordingly, one should predefine a much higher monitoring cost for virtual sensors compared to fixed sensors UAVs to encourage ehysical coverage as much as possible. The parameter $c_{i,t}^{F}(a)$ is used to represent the fail-to-detect cost of event a at location *i* and time *t*, which could cover generalized cost factors sh as (i) response delay in detecting incidents; and (ii) time-dependent traffic incident impacts for different events and (iii) the time duration spent to monitor the traffic impact area.

W assume the preferred departure time and arrival times of UAVs are given without loss of generality. The finial optimization goal in our model is to minimize the monitoring cost of all incidents by using fixed sensors or UAVs in the space-time network, subject to a number of essential constraints such as flow balance constraints for each flight trajectory, budget constraints for both fixed sensors and UAV routing costs.

### 3.2. Space-Time Network Construction

The concept of space-time network (STN) is widely used in both transportation geography and transportation network modeling literature and it aims to integrate physical transportation networks with individual time-dependent movements or trajectories. For more detail about conceptual model of STN, please see the references on Hagerstrand [1], Miller [2] and Kuijpers and Othman [28]. In this paper, we have created the UAV flying ty based on STN.

Ge set N (with a set of incidents A) and physical link set G, the next task is to build the STN structure that can model the network-based UAV trajectory. The steps for building a space-time network for an UAV flying trajectory is shown below:

*Step 1: Build space-time vertex V*
  Add vertex (i, t) to V for i∈N and each *t*.
*Step 2: Build space-time arc set E*
  Step 2.1: Add space-time traveling arc (i,t), (j,t+TT(i,j,t)) to E, for physical link(i,j)∈E, where TT(i, j,t) is the link travel time from node i to node j starting at time t.
  Step 2.2: Add a set of space-time staying/waiting arcs for a pair of vertexes (i,t), (i,t+1) to E, for each time t.

A hypothetic 3-node network is created with time-invariant link travel time in order to illustrate the concept of space-time network construction and reasonable UAV flying trajectory. The detailed information of this hypothetic 3-node network is shown in Table 4.

**Table 4:** Flying time in hypothetic 3-node network.

Link	Travel Time
$\left(o,d_{1}),(d_{1},o\right)$	2
$\left(d_{1},d_{2}),(d_{2},d_{1}\right)$	3

**Figure 5:**
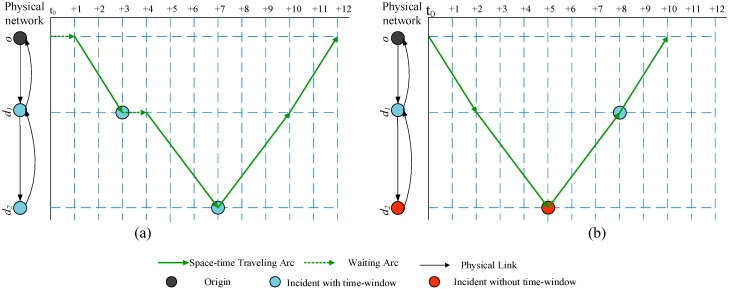
An illustration of flying trajectory space-time network building.

Two cases are shown in Figure 5: (a) two incidents with time-window requirements; and (b) one incident with time-window requirement and one incident without (tight) te-window requirement. Table 5 lists the detailed parameters of incidents for case (a) and case (b), respectively.

**Table 5:** Description of incidents on Figure 5.

Incident Num.	Location of Vertex	Start Time
Case (a)-1	$d_{1}$	3
Case (a)-2	$d_{2}$	7
Case (b)-1	$d_{1}$	8
Case (b)- 2	$d_{2}$	/

First, space-time traveling arcs are constructed only when their corresponding physical links or admissible airspace exist, and the planning time horizon is assumed as 12 time units. We can illustrate how a feasible tour is generated in the space-time network. In case (a) of Figure 5, an UAV first uses waiting arc$\left(o,\;o,t_{0},t_{0}+1\right)$, then uses traveling arc $\left(o,\;d_{1},t_{0}+1,t_{0}+3\right)$ to reach $d_{1}$ and detect no.1 incident. It then uses waiting arc $\left(d_{1},\;d_{1},t_{0}+3,t_{0}+4\right)$ on $d_{1}$ to wait until time $t_{0}+4$, then it will fly to $d_{2}$ through arc $\left(d_{1},\;d_{2},t_{0}+4,t_{0}+7\right)$ to detect incident no. 2. This is followed by a return trip to the departure node *o* through $\left(d_{2},d_{1}\right)$ and $\left(d_{1},o\right)$.

In case (b) of Figure 5, incident 1 occurs at time $t_{0}+8$, and incident 2 is a minor roadside or non-blocking incident without a specific visiting time requirement. An UAV first travels to $d_{2}$ at $t_{0}+5$, then it finishes the monitoring task for no. 1 incident through travelling arc $\left(d_{2},\;d_{1},t_{0}+5,t_{0}+8\right)$. The UAV will go back to the departure node *o* through $\left(d_{1},o\right)$ and arrive *o* at time ${\text{t}}_{0}+10$.

### 3.3. Model Description

We now describe the formal problem statement as follows. The general objectives for the route optimization problem could include information processing cost, operational cost, construction cost. Our model specifically aims to maximize the spatial and temporal coverage for the incident road segments, given the total construction budget of fixed sensors and the UAV operational cost constraints. Thus, the equivalent objective function is to minimize the non-detecting cost.

*Model*:

(1) $$\mathrm{Obj.}\;min\sum_{a\in A,\left(i,t\right)\in \text{ϕ}\left(a\right)}\left[c_{i,t}^{F}(a)\times x_{i,t}^{F}(a)\right]$$

Subject to:

*Event detection constraint:* an event must be detected/virtually detected exactly once.

(2) $$x_{i,t}\left(a\right)+x_{i,t}^{F}\left(a\right)=1\;\forall \left(i,t\right)\in \text{ϕ}\left(a\right)$$

In Equation (2), for the incidents with time windows, if it is detected by fixed sensors or UAVs, then $x_{i,t}\left(a\right)=1$ and $x_{i,t}^{F}\left(a\right)=0$; otherwise, it is covered by the virtual sensors where $x_{i,t}\left(a\right)=0$ and $x_{i,t}^{F}\left(a\right)=1$.

*Eetection and sensors coupling constraint:* an event can be detected at certain space-time vertex, if this vertex is covered by a fixed sensor or UAVs.

(3) $$x_{i,t}\left(a\right)\leq y_{i}+\sum_{f\in F,\text{j},\text{s}}w_{i,j,t,s}\left(f\right),\;forall\;a\in A,\left(i,t\right)\in \text{ϕ}\left(a\right)$$

It should be remarked that in Equation (3), *x_i_*,_*t*_(*a*) is a variable to represent whether the space time vertex (*i*, *t*) in event *a* is detected by an UAV or fixed sensor. If *x_i_*,_*t*_(*a*) = 1, then this space time vertex (*i*, *t*) is detected by an UAV or fixed sensor, that is, $\underset{f\in F,\text{j},\text{s}}{\sum }w_{i,j,t,s}\left(f\right)\;=1$ or *y_i_* = 1. Otherwise, *x_i_*,_*t*_(*a*) = 0 indicates *y_i_* = 0 and $\underset{f\in F,\text{j},\text{s}}{\sum }w_{i,j,t,s}\left(f\right)\;=0$.

*Flow balance constraint:* to depict a time-dependent UAV tour in the space-time network, a set of flow balance constraints is formulated below. This model permits more than one depots exist in the network, and also permits more than one UAVs to execute the mission. We define a super origin vertex and super sink vertex for every UAV, and all vertexes follow the flow balance constraints strictly.

(4) $$\underset{\left(i,j,t,s\right)\in V}{\sum }w_{i,j,t,s}\left(f\right)-\underset{\left(i,j,t,s\right)\in V}{\sum }w_{j,i,s,t}\left(f\right)=\{\begin{array}{l} 1\;\;\;\;\;\;\;i=o^{f},t=EDT^{f}\\ -1\;\;\;i=d^{f},t=LAT^{f}\;\;for\;\;all\;\;f\in F\\ 0\;\;\;\;\;\;\;\;\;\;\;otherwise \end{array}$$

*UAVs’ conflict-free constraint:* each vertex at a specific time can only pass one UAV.

(5) $$\underset{f\in F,\text{j},\text{s}}{\sum }w_{i,j,t,s}\left(f\right)\leq 1\;\forall (i,t)$$

It should be remarked that UAV should satisfy this conflict-free constraint for all the space-time vertex (*i*, *t*).

*Fixed sensor budget constraint:* the total budget should include fix sensor construction cost.

(6) $$\underset{i}{\sum }y_{i}d_{i}\leq B$$

*UAV operational constraint:* the maximum fly distance or flying time constraint for each UAV.

(7) $$\underset{\left(i,j,t,s\right)\in E}{\sum }d_{i,j,t,s}\times w_{i,j,t,s}\left(f\right)\leq K(f)\;\;\;\;\;\;\forall f\in F$$

Without the loss of generality, our paper considers the total flying time. In Equation (7), $d_{i,j,t,s}$ is fixed for each road segment. There are also binary definitional constraints for variables $x_{i,t}(a)$, $x^{F}\left(a\right)$, $y_{i}$ and $w_{j,i,s,t}\left(f\right)$.

$$\begin{array}{l} x_{i,t}(a)\in \left\{0,1\right\},\;\mathrm{for}\;\mathrm{all}\;a\in A,\;i\in N\\ x_{i,t}^{F}\left(a\right)\in \left\{0,1\right\},\;\mathrm{for}\;\mathrm{all}\;a\in A,\;i\in N\\ y_{i}\in \left\{0,1\right\},\;\mathrm{for}\;\mathrm{all}\;i\in N\\ w_{j,i,s,t}\left(f\right)\in \left\{0,1\right\},\;\mathrm{for}\;\mathrm{all}(j,i,s,t)\in E,\;f\in F \end{array}$$

### 3.4. Model Simplification

To derive a simple form of the model, we first substitute Equation (2) into the objective function and obtain the following objective function in Equation (8).

(8) $$\min\sum_{a\in A,\left(i,t\right)\in \text{ϕ}\left(a\right)}\left[c_{i,t}^{F}(a)\times (1-x_{i,t}(a))\right]$$

In practice, after years of incident detection system construction, fixed sensors have been equipped at important locations in highway networks. Accordingly, in our model the location of fixed sensors are assumed in advance, and the corresponding variable *y_i_* equals to 1 at location *i* where a fixed sensor is allocated. Then $x_{i,t}\left(a\right)$ = 1 for *y_i_* = 1, so we can construct ϕ′(a) to exclude the space time vertexes covered by the fixed detectors.

Without loss of generality, we use the time unit (min) as the generalized cost unit so that we can minimize the monitoring cost of all incidents. One can further use the value of time as the coefficient to convert the different degree of non-detection to generalized monetary costs, in conjunction with the other system costs involving fixed sensor operations and UAV energy costs typically expressed as a function of UAV flight distance and speed.

According to Equation (5), any two UAVs cannot arrive at a same space-time vertex (*i*, *t*) to avoid conflicts, which means that $\underset{f\in F,\text{j},\text{s}}{\sum }w_{i,j,t,s}\left(f\right)$ can only equal to 1 or 0 this case, if $\underset{f\in F,\text{j},\text{s}}{\sum }w_{i,j,t,s}\left(f\right)$ = 1 at space-time vertex (*i,t*), then $x_{i,t}\left(a\right)$ = 1; otherwise, if $\underset{f\in F,\text{j},\text{s}}{\sum }w_{i,j,t,s}\left(f\right)$ = 0, $x_{i,t}\left(a\right)$ = 0. Thus, we can derive Equation (9) as:

(9) $$x_{i,t}\left(a\right)=\underset{f\in F,\text{j},\text{s}}{\sum }w_{i,j,t,s}\left(f\right)\;\;for\;all\;a\in A,\left(i,t\right)\in \text{ϕ}\left(a\right),y_{i}=0$$

and then obtain the simplified model with optimization function Equation (10):

(10) $$\min\sum_{a\in A,\left(i,t\right)\in \text{ϕ}\prime \left(a\right)}\left\{-c_{i,t}^{F}(a)\times \underset{f\in F,j,s}{\sum }w_{i,j,t,s}\left(f\right)\right\}$$

Subject to, Equations (4), (5) and (7) and the binary variable definitional constraints.

## 4. Lagrangian Relaxation-Based Solution Algorithms

The Lagrangian relaxation technique is commonly used for solving optimization problems that contain “hard” constraints. Compared to the primal problem, the relaxation problem can often be diverted/decomposed to classic or easy-to-solve sub-problems.

### 4.1. Lagrangian Function

As constraints in Equations (5) and (7) are considered as hard constraints, they are further relaxed by introducing two sets of multipliers, namely, non-conflict multiplier ${\text{β}}_{i,\text{t}}$ and UAV budget multiplier μ(f). The Lagrangian relaxation function $L_{w(f)}$ can be defined in Equation (11) subject to the flow balance constraint (4). For each UAV, the problem can simplified as time-dependent least-cost path sub-problem:

(11) $$L_{w(f)}=\sum_{a\in A,\left(i,t\right)\in \text{ϕ}\prime \left(a\right)}\left\{-c_{i,t}^{F}(a)\times \sum_{j,s}w_{i,j,t,s}\left(f\right)\right\}+\sum_{f}\text{μ}(f)\times \left[\underset{\left(i,j,t,s\right)\in E}{\sum }d_{i,j,t,s}\times w_{i,j,t,s}\left(f\right)-K(f)\right]+\sum_{i,t}\left[{\text{β}}_{i,t}\times \left(\sum_{j,s}w_{i,j,t,s}\left(f\right)-1\right)\right]=\underset{\left(i,j,t,s\right)\in E}{\sum }\left[c\prime_{i,j,t,s}\times w_{i,j,t,s}\left(f\right)\right]-\sum_{f}\text{μ}(f)\times K(f)-\sum_{i,t}{\text{β}}_{i,t}$$

where, the generalized cost term $c\prime_{i,j,t,s}$ = $\text{μ}(f)\times d_{i,j,t,s}+{\text{β}}_{i,t}-\sum_{a}\left\{{\text{θ}}_{i,t}^{a}\times c_{i,t}^{F}(a)\right\}$. Parameter ${\text{θ}}_{i,t}^{a}$ = 1, if (i,t) is included in event set a. $\text{μ}(f)\times d_{i,j,t,s}$ reflects the use of fuel, and if the total energy is insufficient, then μ(f) has to be increased to penalize fuel-inefficient routes.

${\text{β}}_{i,t}$ reflects the potential conflict between flights. If there are more than 2 flights at the same space-time vertex, then ${\text{β}}_{i,t}$ needs to be increased to prevent the conflict.

Overall, $\sum_{a}\left\{{\text{θ}}_{i,t}^{a}\times \left[c_{i,t}(a)-c_{i,t}^{F}(a)\right]\right\}$ reflects the benefit collected by flight routing plan, e.g., early detection and complete space-time coverage for the entire event, as well as potential loss due to non-detected space-time points.

### 4.2. Solution Procedure

In this section, we further explain the optimization algorithm for solving Lagrangian relaxation-based problem $L_{w(f)}$.

Step 1. Initialization

Set iteration number *m* = 0;

Choose positive values to initialize the set of Lagrangian multipliers μ(f), ${\text{β}}_{i,t}$;

Step 2. Solve simplified problems

Solve subproblem $L_{w(f)}$ using a standard time-dependent least cost path algorithm and find a path solution for each UAV *f*.

Calculate primal, and gap values of $L_{w(f)}$;

Step 3. Update Lagrangian multipliers

Update Lagrangian multipliers μ(f), ${\text{β}}_{i,t}$ using subgradient method;

Step 4. Termination condition

If *m* is larger than a predetermined maximum iteration value, or the gap is smaller than a previously given toleration gap, terminate the algorithm, otherwise *m = m + 1* and one must go back to Step 2.

To generate the upper bound feasible solutions, one can also use a Lagrangian heuristic algorithm by iteratively fixing the feasible routing solution for individual vehicles, while the adjusted multipliers from the lower bound solutions can be used to guide the iterative refinement of the generalized cost for the UAV routing problem. For detailed procedures on time-dependent shortest path algorithms and sub-gradient updating rules in a Lagrangian relaxation solution framework, we refer interested readers to the study by Meng and Zhou [29] for train routing and scheduling applications.

## 5. Numerical Experiments

This section evaluates the results of the UAV route planning under different conditions, which aims to demonstrate the effectiveness of the proposed method in the context of real-world networks.

### 5.1. Simple Illustrative Example

**Figure 6:**
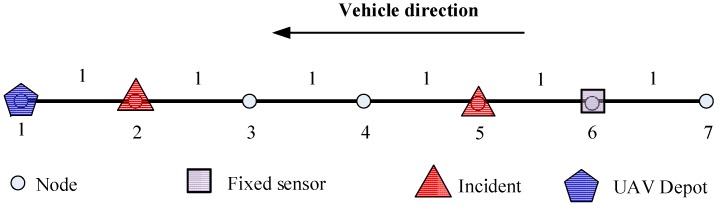
Demonstration of UAV cruise route.

Using the simple demonstrative network in Figure 6, we want to illustrate how the proposed model can effectively plan UAV routes.

In this example, the UAV depot is located at node 1 and the fixed sensor is installed at node 6. The flying time of UAV for every two successive nodes are 1 time unit and the total allowed flying time duration is 16 time unit. The UAV can be deployed after time 1.

**Figure 7:**
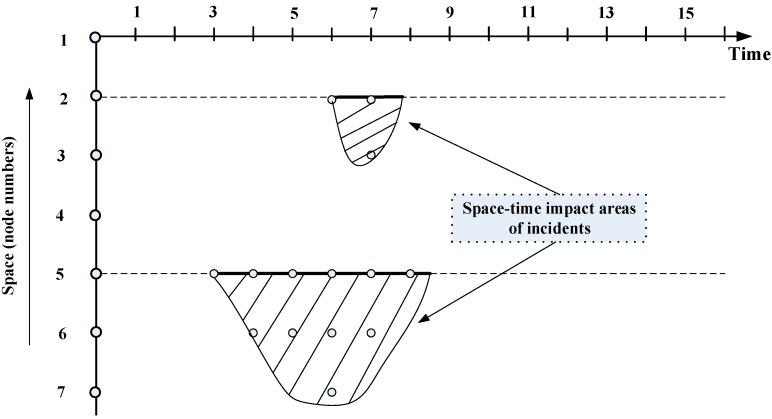
Space-time impact area of these two incidents with first time window of 6–8 and second time window of 3–8.5.

Assume there is only one UAV and two incidents on this network. No. 1 incident on node 2 starts at time 6 and ends at time 8, and it also propagates to node 3 at time 7. No. 2 incident affects node 5, 6 and 7 and the duration for each nodes are time 3 to time 8.5, time 4 to time 7 and time 6 respectively. The space-time influence area of these two incidents is shown below in Figure 7.

**Figure 8:**
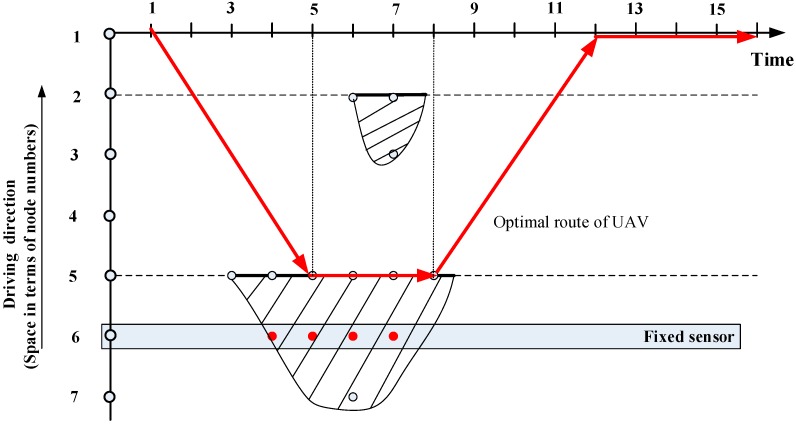
Optimal route of UAV from GAMS with a total flying time constraint of 15 time units.

The UAV’s route planning problem is implemented and solved by a commercial solution solver, General Algebraic Modeling System (GAMS) using the model we developed in previous sections. The optimal routing plan of UAV is shown in Figure 8, where the vertical axis shows the space dimension with the horizontal axis for the time dimension. Given the total flying time constraint of 15 time units, the optimal route of UAV for this example is: node 1 (time 1) → node 2 (time 2) → node 3 (time 3) → node 4 (time 4) → node 5 (time 5–time 8) → node 4 (time 9) → node 3 (time 10) → node 2 (time 11) → node 1 (time 12–time 16). Due to the flying time constraint, the optimal solution cannot cover No. 1 incident, and the UAV arrives at node 5 after time 2, since No. 2 incident start and it does not reach to node 6 and node 7, while node 6 is covered by the fixed sensor.

### 5.2. Medium-Scale Experiments

**Figure 9:**
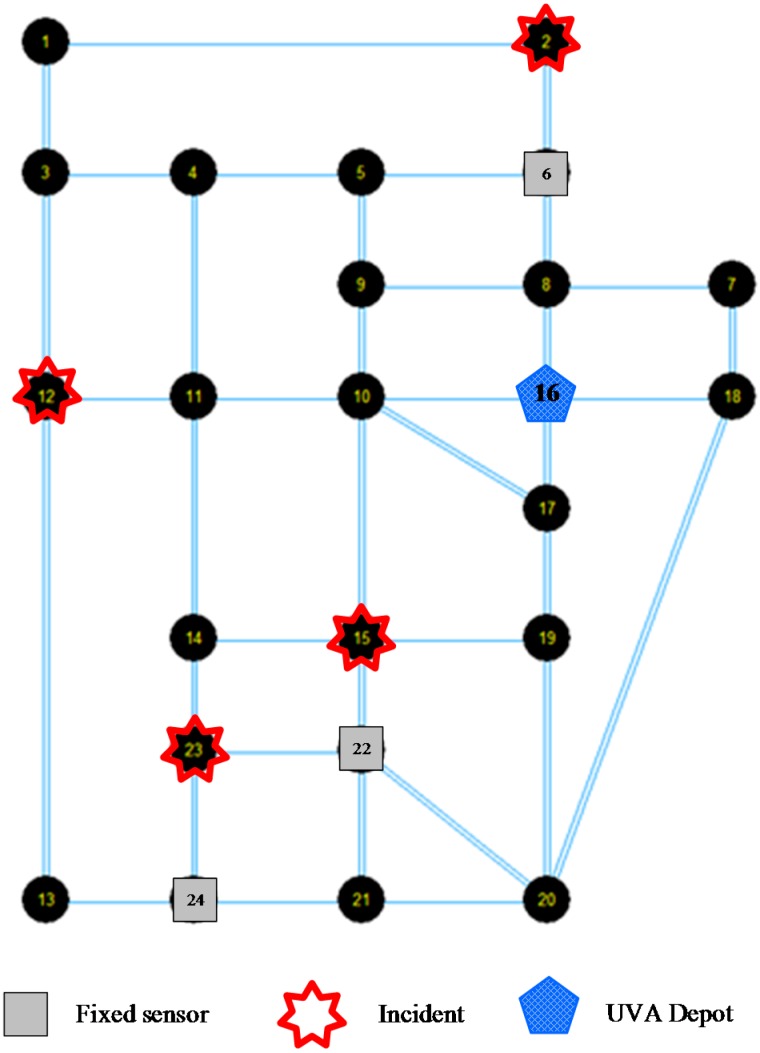
Simplified Sioux Falls network.

A simplified Sioux Falls network consisting of 24 nodes and 76 directional links is shown in Figure 9. We assume the depot of UAV is located at node 16, and fixed sensors are installed at nodes 6, 22 and 24. There is one available UAV for this network and the total allowed flying time duration is 500 min. The flying time durations of the UAV on each road are shown in Table 6. The optimization model is solved on a personal computer with an Intel i7-3630 QM 2.4GHz CPU and 16 GB RAM.

Four incidents are assume to occur on this network on nodes 2, 12, 15 and 23. The detailed propagation and duration time for each incident are listed in Table 7.

**Figure 10:**
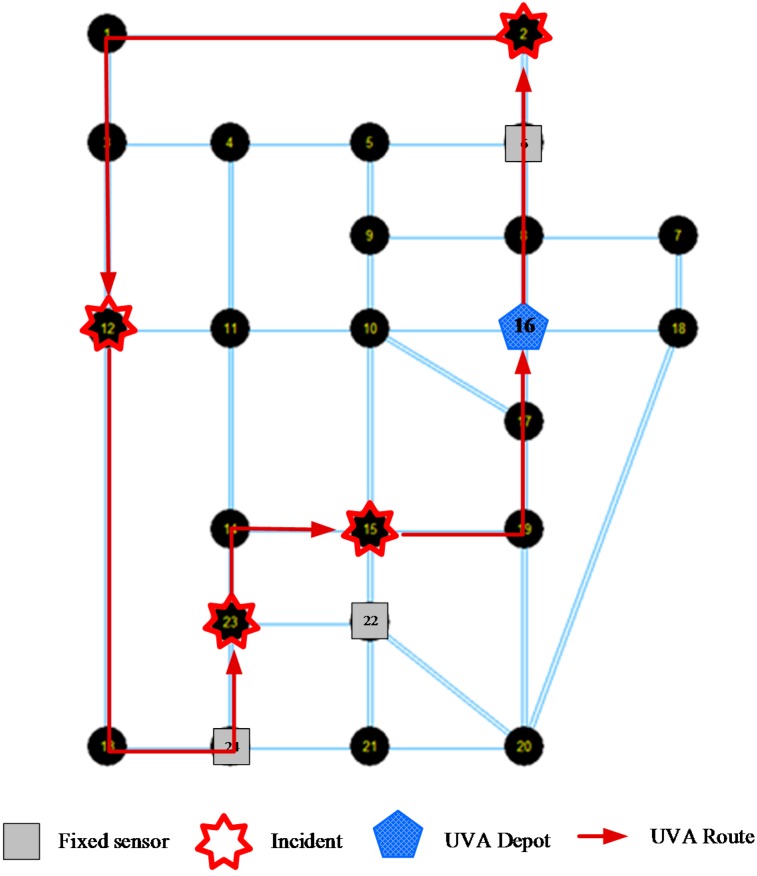
Optimal UAV cruise route.

There are a total of 161 space-time vertexes covered by these four incidents. Since there are fixed sensors on nodes 6, 22 and 24, thus 46 space-time vertexes of incidents can be detected by fixed sensors. The other 161 − 46 = 115 space-time vertexes of incidents will be considered to be covered by UAV routes. The optimal UAV route is shown in Figure 10.

The optimal UAV cruise route is: node 16 (Depot, 1–76 min) → node 8 (86 min) → node 6 (90 min) → node 2 (incident 1, 100–112 min) → node 1 (124 min) → node 3 (132 min) → node 12 (incident 2, 140–166 min) → node 13 (172 min) → node 24 (180 min) → node 23 (incident 4, 185–212 min) → node 14 (220 min) → node 15 (incident 3, 230–245 min) → node 19 (251 min) → node 17 (255 min) → node 16 (depot, 259–500 min). In this optimal UAV route, 83 space-time vertexes are detected by the UAV, while the others are undetected.

**Table 6:** UAV flying time on all links.

From Node	To Node	Flying Time (min)	From Node	To Node	Flying Time (min)	From Node	To Node	Flying Time (min)	From Node	To Node	Flying Time (min)
1	2	12	8	7	6	13	24	8	19	17	4
1	3	8	8	9	20	14	11	8	19	20	8
2	1	12	8	16	10	14	15	10	20	18	8
2	6	10	9	5	10	14	23	8	20	19	8
3	1	8	9	8	20	15	10	12	20	21	12
3	4	8	9	10	6	15	14	10	20	22	10
3	12	8	10	9	6	15	19	6	21	20	12
4	3	8	10	11	10	15	22	6	21	22	4
4	5	4	10	15	12	16	8	10	21	24	6
4	11	12	10	16	8	16	10	8	22	15	6
5	4	4	10	17	16	16	17	4	22	20	10
5	6	8	11	4	12	16	18	6	22	21	4
5	9	10	11	10	10	17	10	16	22	23	8
6	2	10	11	12	12	17	16	4	23	14	8
6	5	8	11	14	8	17	19	4	23	22	8
6	8	4	12	3	8	18	7	4	23	24	4
7	8	6	12	11	12	18	16	6	24	13	8
7	18	4	12	13	6	18	20	8	24	21	6
8	6	4	13	12	6	19	15	6	24	23	4

**Table 7:** Incidents information.

	Incident 1	Incident 2	Incident 3	Incident 4
Start node	2	12	15	23
Start time (min)	100	140	230	185
Space-time impact area (node, duration)	2, (100–125) 6, (121–130)	12, (140–165) 13, (160–165)	15, (230–245) 22, (238–247) 21, (245–249)	23, (185–212) 24, (200–225) 21, (222–225)

**Figure 11:**
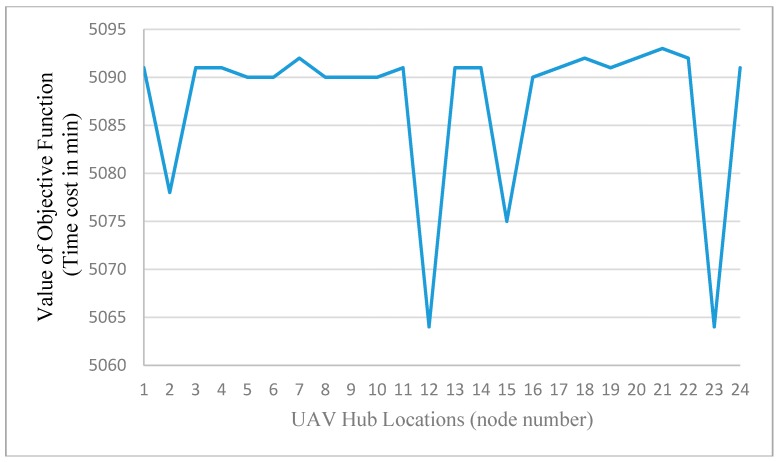
Sensitivity analysis of different UAV’s depots.

A quick sensitivity analysis has been also performed by varying the UAV airbase locations on different nodes. The total cost are used as the evaluation index, and the results shown in Figure 11 indicates that the locations at nodes 12, 15, and 23 are more advantageous. As those locations are coincident with the presumed incident locations, so the observations from Figure 11 are expected for this medium scale network. However, the results also indicate that we need to select the UAV depot location more carefully and systematically to minimize the total unexpected cost. The objective value in Figure 11 corresponds to the total penalty cost for undetected space-time vertexes (by the fixed sensors or UAVs). Without loss of generality, we use the time unit (min) as the generalized cost unit. One can further use the value of time as the coefficient to convert the degree of non-detection to generalized monetary costs, in conjunction with the other system costs involving operations and energy costs.

### 5.3. Chicago Networks

**Figure 12:**
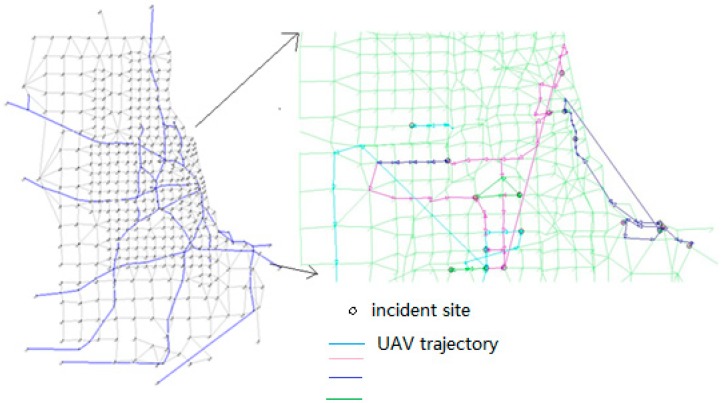
Left: Chicago sketch network with 933 nodes and 2950 links; right: Sameple UAV routing map in the subarea with circles representing incident sites and color lines representing flight routes for four different UAVs.

The above results are provided from a standard optimization solver, which has difficulties in solving large-scale instances on real-world regional networks. Obviously, if we can implement the time-dependent least cost shortest path algorithm directly in high-performance programming languages such as C++ or Fortran, then the proposed Lagrangian relaxation solution procedure can better handle the most computational consuming step for large-scale applications. To this end, we implemented the proposed Lagrangian relaxation algorithm in C++, and test the computational performance of the proposed algorithm on the large-scale Chicago sketch network with 933 nodes, shown in the left plot of Figure 12. In this example, we consider 2 h as the planning horizon (*i.e*., 120 time intervals with 1 min as temporal resolution), and then randomly generate incident locations. The impact of incidents is simulated through an open-source dynamic traffic assignment simulator [30], DTALite using the time-dependent traffic origin-destination demand tables with reduced capacity due to incidents.

**Figure 13:**
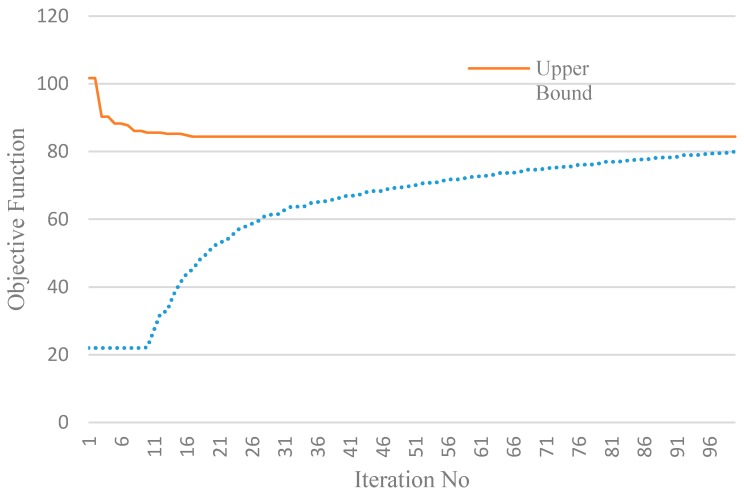
Evolution of Lagrangian relaxation-based upper and lower bound series in the Chicago sketch network with four UAVs and 20 incidents; the lower bound value is generated using Equation (11) and the upper bound is generated by converting possibly infeasible routing solution to satisfy all constraints.

The right plot of Figure 12 demonstrates a sample UAV routing with four UAVs, which could help readers understand the complexity of the problem solved. First, we consider 20 randomly generated incident sites with four UAVs to be scheduled. Figure 13 shows the evolution of lower bound (LB) and upper bound (UB) values in the first 100 iterations, which converges to a significantly small relative solution gap of 5.09%, defined as (UB-LB)/UB. We also observe that, the solution quality gap between upper bound and lower bound starts to reduce steadily after the first 10–15 iterations, as the subgradient algorithm needs to take a few iterations to approximate Lagrangian multipliers with reasonable values. The slow converging pattern afterwards can be explained by the relatively small step size used in the subgradient algorithm when iterative algorithm further proceeds.

**Figure 14:**
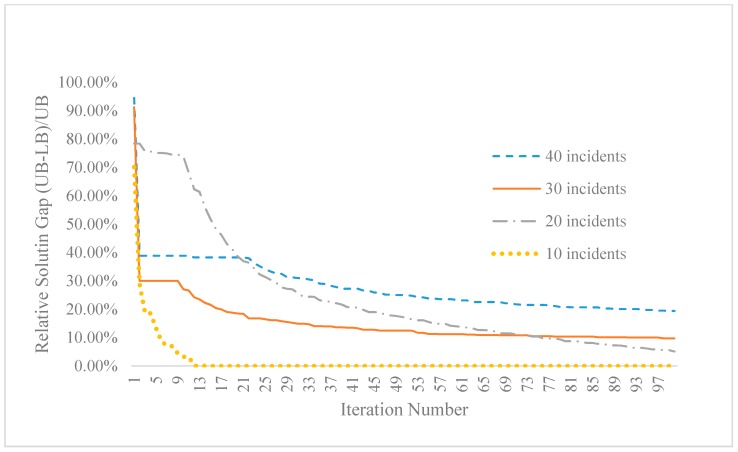
Convering patterns of relative solution gap for four UAVs with different numbers of incidents in Chicago sketch network.

Figure 14 further shows the relative solution gap for different numbers of randomly generated incidents, namely 10, 20, 30 and 40 for the same given four UAVs. A small case of 10 incidents converges the optimal solution with the gap of 0% within 20 iterations. When there are a large number of locations to be covered, the algorithm results in larger solution gaps with a slower converging pace. Specifically, 30 and 40 incidents lead to relative solution gaps of 9.7% and 19.4%, respectively, and there are about 8 out of 30 and 19 out of 40 incidents which cannot be covered by UAVs at all. When the number of locations to be covered increases but still with limited UAV resources, the proposed approximation algorithm has to handle the complexity in determining the trade-off of visiting different sites with constrained space-time prism. Overall, the large solution gap is introduced by additional complexity introduced by the number of space-time locations and corresponding LR multipliers. On the other hand, we need to still recognize both theoretical and practical value of the Lagrangian solution algorithm, as it can produce results with exact guarantee on solution accuracy (say 5% or 20%), and the generated upper and lower bound solutions further provide the guidance and benchmark for heuristic algorithms to find close-to-optimal solutions within computational budget.

**Figure 15:**
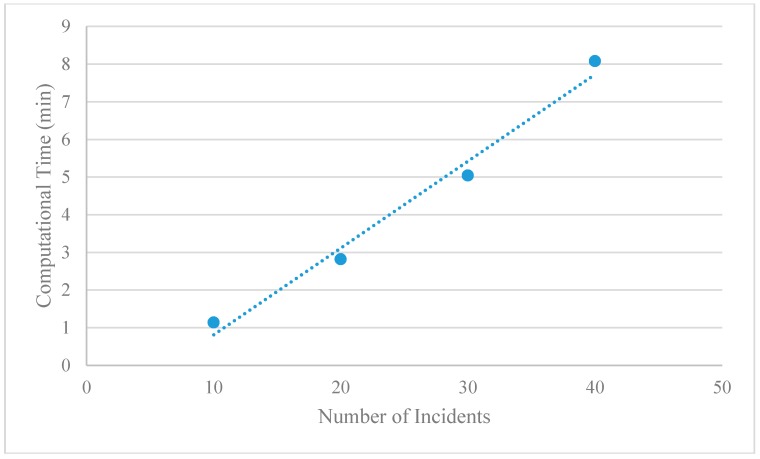
Computaional time with different numbers of incidents in Chicago sketch network.

The computational time of the proposed solution increases almost linearly with an increase of incident numbers, shown in Figure 15. In general, when there are many incidents to be covered, the number of Lagrangian multipliers in Equation (11) also increases, which could require a significant amount of additional computational time for the time-dependent least cost shortest path algorithm to find the optimal solutions in the proposed space-time network. The whole search process with 100 iterations typically takes an average of 1 to 10 CPU min under different numbers of incidents, while the a single iteration of the LR-based lower bound and upper bound generation uses an average of 28.19 milliseconds for the case of 20 incidents. As expected, the most significant amount of time has been spent for constructing a space-time path in the test network with 2950 links and 120 time intervals.

## 6. Conclusions and Future Research Plan

The fixed sensor-oriented traffic sensor network design problem has been widely studied. With UAVs as a special type of mobile sensors, this paper aims to develop a practically useful and computationally efficient mobile sensor routing model for non-recurring and recurring traffic state detection. Based on the time geography perspective, we present a linear integer programming model to maximize spatial and temporal coverage of traffic state detection under various UAV speed, admissible airspace and operational budget constraints. A Lagrangian relaxation solution framework is developed to effectively simplify the original complex problem into standard time-dependent least cost path problems. Using a number of illustrative and real-world networks, our proposed model offers a unified fixed and mobile sensor network framework and efficient routing/scheduling algorithms for improving road network observability.

With special focus on mobile sensors on daily operations, this paper considers the fixed sensor locations as predefined parameters. In our future research, we will jointly optimize the fixed sensor locations and UAV’s route planning under a large number of random traffic conditions, within a stochastic optimization modeling framework. By doing so, it would be interesting to analyze the cost-benefit between mobile and fixed sensors in order to establish a mutually complementary and fully integrated sensor network. Our further research will be also focused on the efficient exact and heuristics algorithms for real-time UAV routing algorithms with unknown or predicted traffic conditions.
